# Association of monocyte/high-density lipoprotein cholesterol ratio and the carotid intima-media thickness in diabetic patients

**DOI:** 10.1186/s12902-022-01246-6

**Published:** 2022-12-19

**Authors:** Atefeh Amouzegar, Zahra Mirzaasgari, Ali Mehrabi, Mojtaba Malek, Fariba Alaei-Shahmiri, Laily Najafi, Alireza Khajavi

**Affiliations:** 1 Department of Nephrology, Endocrine Research Center, Institute of Endocrinology and Metabolism, Iran University of Medical Sciences (IUMS), Tehran, Iran; 2 Firoozgar Clinical Research Development Center, School of Medicine, Iran University of Medical Sciences, Tehran, Iran; 3 Department of Neurology, Firoozgar Hospital, School of Medicine, Iran University of Medical Sciences, Tehran, Iran; 4 Firoozgar Clinical Research Development Center (FCRDC), Iran University of Medical Sciences, Tehran, Iran; 5 Research Center for prevention of cardiovascular disease, Institute of Endocrinology and Metabolism, Iran University of Medical Sciences (IUMS), Tehran, Iran; 6 Endocrine Research Center, Institute of Endocrinology and Metabolism, Iran University of Medical Sciences (IUMS), 3rd floor, No10, Firouzeh alley, South Vali-Asr Ave., Vali-Asr Sq, Tehran, Iran; 7 Student Research Committee, Faculty of Paramedical Sciences, Shahid Beheshti University of Medical Sciences, Tehran, Iran

**Keywords:** Association, Monocyte/high-density lipoprotein cholesterol ratio, Carotid intima-media thickness, Type 2 diabetes mellitus

## Abstract

**Objectives:**

The goal of this study was to see whether there was a link between the monocyte/high-density lipoprotein cholesterol ratio (MHR) and carotid intima-media thickness (CIMT) in people with type 2 diabetes.

**Methods:**

Duplex ultrasonography parameters and demographic, physical, and paraclinical assessments were recorded. Using the t-test, the MHR and CIMT were compared between the two groups. Regression models were also constructed.

**Results:**

A total of 118 diabetics and 126 non-diabetics were included in the cross-sectional research. According to the stated diabetes duration, the observed age difference of 7 years might be considered. The MHR and CIMT were not substantially different between the two groups. In the DM and non-DM groups, the Spearman correlations between MHR and CIMT were 0.32 and − 0.08, respectively (*p*-values = 0.001 and 0.379). Thus, regression models (stratified for DM/non-DM and male/female) revealed that the MHR is a significant predictor of CIMT, but only in the case of male DM individuals, when crudely adjusted for confounders.

**Conclusions:**

In diabetes mellitus, the current investigation found a direct link between MHR and CIMT. In addition, in male diabetic subjects, MHR was demonstrated to be a predictor of CIMT.

## Introduction

Patients with type 2 diabetes mellitus are more likely to develop macrovascular complications of diabetes, such as cardiovascular disease (CVD) and atherosclerosis (AS), which are mostly due to dyslipidemia (abnormal glucose and lipid metabolism), as well as a chronic inflammatory state [1–4]. Inflammation solely may be considered a systemic process that increases inflammatory mediators, and not just a local reaction [3]. During systemic inflammation and atherogenesis, macrophages and monocytes are the most prominent sources of proinflammatory and pro-oxidant cytokines [5]. In AS, macrophages and monocytes remove modified and oxidized low-density lipoproteins (LDLs), which are then attracted into the artery wall, causing the release of inflammatory cytokines in inflamed tissue. As a result, inflammatory cholesterol ester-loaded plaque forms [6]. The number of circulating monocytes is a predictor of new plaque development [7]. High-density lipoprotein cholesterol (HDL-C), on the other hand, has antiatherosclerotic properties by neutralizing monocytes’ proinflammatory and pro-oxidant actions by decreasing macrophage migration and LDL oxidation, as well as cholesterol export from these cells [8]. HDL-C has also been found to protect endothelial cells from the negative effects of LDL-Cholesterol (LDL-C) [9]. Furthermore, HDL-C inhibits monocyte accumulation as well as the proliferation and differentiation of monocyte progenitor cells. As a result, monocyte accumulation and HDL-C decrease may play a role in AS and CVD. Given that inflammation and oxidative stress are the primary causes of inflammatory diseases [10], many other studies have focused on determining whether the Monocyte/HDL-C ratio (MHR) could be a useful marker for predicting the development and progression of inflammatory processes such as AS, CVD, or slow coronary flow, in addition to the relationship between a high number of monocytes and low HDL-C levels that has been reported in inflammatory disorders [3, 10]. MHR is a new and easily available metric for determining inflammatory and oxidative stress levels in the body [11]. MHR has been reported to be linked to cardiovascular (CV) events in individuals with chronic kidney disease (CKD) in earlier investigations [12]. The presence of slow coronary flow is also associated with a higher MHR [10]. Furthermore, elevated MHR was shown to be correlated to diabetic nephropathy, making it a biomarker for diabetic nephropathy [13]. Renal impairment was related to a higher circulating monocyte count and a lower HDL-C content in the blood, as well as more severe AS [14]. The most frequent cause of CKD in the United States and throughout the world is diabetes mellitus (DM), and most of the CKD patients are diagnosed at early stages (stage 1 or 2) [15]. CIMT (carotid intima-media thickness) is a simple and inexpensive test for the diagnosis of preclinical AS [1]. Bots et al. showed that CIMT had prognostic value for later cardio/cerebrovascular events [16]. The goal of this study was to see whether there was a link between the monocyte/high-density lipoprotein cholesterol ratio and carotid intima-media thickness in people with type 2 diabetes as a considerable gap, due to the importance of macrovascular diabetes complications. Furthermore, the other goal is to investigate the influence of chronic inflammation on AS, which is common in DM.

## Materials and methods

### Study design and participants

During 2019–2021, this cross-sectional research done at Endocrine Research Center and Firoozgar teaching tertiary hospital in Tehran, Iran, recruited 244 individuals by convenience sampling. The research protocol (IR.IUMS.REC.1397.1118) was approved by the ethics committee of the Iran University of Medical Sciences, and all participants signed and gave written informed permission.

With completely measured outcomes and variables of relevance, the present research was shown on 118 diabetes and 126 non-diabetic patients. The sample size was determined based on the effect size of 0.36 reported by [17], α = 0.05, and power = 0.8, using the GPower software.

T2DM (DM group), the age range of 30–60 years old, and diabetes duration of more than 5 years were among the inclusion criteria. The exclusion criteria for both groups included smoking and drug abuse, pregnancy, using the corticosteroid, immunosuppressants, omega-3, lipid-lowering agents and contraceptives, renal transplantation, systemic diseases (CVD, autoimmune disease, Cushing, adrenal hyperplasia, chronic lung or renal disease, chronic or acute infection), CV surgery, malignancy, thrombocytopenia, albuminuria, hemoglobin A1c (HbA1C) ≥ 9, LDL-C ≥ 100, triglyceride ≥250, blood pressure (BP) ≥ 160/100, estimated glomerular filtration rate (e-GFR) < 30 and body mass index (BMI) ≥ 35.

### Clinical measurements and definitions

The American Diabetes Association (ADA) guideline 2019 for the DM group was used to determine DM diagnosis [18]. A qualified physician conducted a face-to-face interview at the initial appointment. All participants had their demographics, medical histories, lifestyles, and pharmacological factors collected, as well as a physical examination. Standing height was determined using a calibrated stadiometer (Seca gmbh& co. kg. Germany) and weight was determined using a calibrated digital scale (Seca gmbh& co. kg. Germany). In a controlled environment, blood pressure was monitored using an electronic monitor (Riester, Exacta 1350, Germany) (seated position, after 10 minutes of resting, drinking tea or coffee, and eating food for at least half an hour). After an overnight fast, two samples of the blood clot and EDTA anticoagulated tubes were used to examine the biochemistry panel, which included complete blood count (CBC) analysis (with differential evaluation), fasting blood sugar (FBS), Creatinine, and lipid profile. Using a standard kit, the samples were measured using the Enzymatic Calorimeter technique (Biorex). Weight / height^2^ was used to compute BMI (kilograms per meters squared). The monocyte count was evaluated by applying data provided by the CBC differential analysis. The reference value for monocyte is 2–10%. The MHR was computed for both groups by monocyte counts (× 10^6^/L)/HDL-C (mg/dL).

Moreover, e-GFR was computed by applying the modification of diet in renal disease (MDRD) formula [19].

### Assessment of carotid intima-media thickness (CIMT)

The CIMT was evaluated by a single professional and experienced neurologist with a license in the area of neurosonology who was blinded to the characteristics of all participants. A duplex ultrasound system (B-Mode) with an 8-Hz linear probe (Sonosite M Turbo, Fuji Film, Japan) was used to measure CIMT. A section of the common carotid artery (CCA) was discovered to be free of atherosclerotic plaque. The mean CIMT was calculated by estimating the thickness of the innermost two layers of intima-media in 10 mm before the bifurcation of CCA, where no atherosclerotic plaques were present, according to the procedure. The average of the right and left CIMT was employed in the study. Mean CIMT over the 75th percentile for age, race, and gender was recognized as a risk factor for CV events by the American Echocardiographic Association [20].

### Statistical analysis

The mean standard deviation (SD) was utilized to characterize the normally distributed continuous variables, and the t-test was employed to compare them between the DM and non-DM groups. Furthermore, non-normally distributed continuous variables are given as median (interquartile range (IQR)) and compared between two groups using the non-parametric Mann-Whitney U-test. Furthermore, categorical variables are given as a percentage (%) and the Chi-squared test is used to compare groups. Finally, the MHR was fitted crudely in a linear regression model as a predictor of CIMT and adjusted for factors that were statistically or clinically relevant. A significance level of .05 was chosen. Stata was used to conduct the statistical analysis (ver. 13).

## Results

In the research 118 diabetic and 126 non-diabetic individuals were included, with demographic, physical, and laboratory tests, as well as duplex ultrasonography parameters, all of which were comprehensively assessed. The duration of diabetes is 7 [5–10] years. Table 1 summarizes the demographic characteristics of the participants.

**Table 1:** Demographic characteristics of the study groups (DM and non-DM patients)

Baseline characteristics	DM^†^ (*n* = 118)	Non-DM(*n* = 126)	*P*-value
Age, years	50.31 (9.59)	42.71 (9.82)	< 0.01*
Male gender, n (%)	56 (47.46%)	57 (45.24%)	0.73
Body mass index, kg/m^2^	26.38 (4.56)	25.86 (5.39)	0.42
History of cardiovascular disease, n (%)	22 (18.64%)	28 (22.22%)	0.49
History of Hypertension, n (%)	33 (27.97%)	28 (22.22%)	0.30
History of Hyperlipidemia, n (%)	26 (22.03%)	18 (14.29%)	0.12
Systolic BP^‡^, mmHg	125.1 (12.8)	117.3 (12.1)	< 0.01*
Diastolic BP, mmHg	80.1 (9.2)	75.5 (12.1)	< 0.01*

Data are expressed as mean (SD) for continuous variables and percentage for categorical variables. The p-values were obtained from the t-test and Chi-squared test for continuous and discrete variables

^†^*DM* Diabetes mellitus

^‡^*BP* Blood pressure

* indicates significance

The observed age difference of 7.6 years could be considered in accordance with the reported diabetes duration of 7 years.

Moreover, the family histories of DM and hypertension were considerably different between diabetes and non-diabetes groups (76.3% vs. 38.9 and 72.9% vs. 40.5%) (*P*-value< 0.01). Thus, the family history of the cerebrovascular accident did not differ significantly between the two groups (22.9% vs. 21.5%) (*P*-value = 0.38).

Table 2 also shows a comparison of laboratory parameters between diabetes and non-diabetic groups.

**Table 2:** Comparison of laboratory parameters between diabetes and non-diabetes groups

Laboratory variables	DM^†^ (*n* = 118)	Non-DM (*n* = 126)	*P*-value
Fasting blood sugar, mg/dl	115(103–138)	96(92–103)	< 0.01*
Creatinine, mg/dl	0.9(0.8–1)	0.9(0.7–1)	0.06
Total cholesterol, mg/dl	132(122–146)	149(123–182)	< 0.01*
Triglyceride, mg/dl	105(89–124)	95(75–148)	0.29
HDL-C^α^, mg/dl	44(41–48)	43(41–48)	0.57
LDL-C^β^, mg/dl	70(58–83)	95(80–118)	< 0.01*
WBC^μ^ count, × 10^9^/L	5800(5100–7000)	6200(5300–7500)	0.09
Monocyte count×10^9^/L	2(2–3)	2(2–3)	0.35
Hemoglobin, gr/dl	13.38 (1.45)	14.22 (1.93)	< 0.01*
e-GFR^¥^	95.75 (31.71)	108.35 (34.35)	< 0.01*

Data are expressed as mean (SD) and median (interquartile range) for normally and non-normally distributed variables, applying the t-test and Mann-Whitney test, respectively

^†^*DM* Diabetes mellitus

^α^*HDL-C* High-density lipoprotein cholesterol

^β^*LDL-C* Low-density lipoprotein cholesterol

^μ^*WBC* White blood cell

^¥^*e-GFR* Estimated glomerular filtration rate

* indicates significance

Table 2 indicates lower levels of cholesterol, LDL, hemoglobin, and e-GFR among DM patients.

Table 3 compares the variables of concern, MHR, and CIMT, across diabetes and non-diabetic groups. The median test yielded the indicated *p*-values.

**Table 3:** Comparison of monocyte/high-density lipoprotein cholesterol ratio and carotid intima-media thickness between diabetes and non-diabetes groups

Variables	DM^†^ (*n* = 118)	Non-DM (*n* = 126)	*P*-value
MHR^α^	2.65(1.87–4.37)	3.14(2.18–4.93)	0.13
RT CIMT^β^	0.4(0.3–0.5)	0.4(0.3–0.5)	0.85
LT CIMT^μ^	0.4(0.3–0.5)	0.4(0.3–0.5)	0.60
CIMT ^γ^	0.4(0.35–0.5)	0.4(0.35–0.5)	0.56

Data are expressed as median (interquartile range), applying the Mann-Whitney test

^†^*DM* Diabetes mellitus

^α^*MHR* Monocyte/high-density lipoprotein cholesterol ratio

^β^*RT CIMT* Right carotid intima-media thickness

^μ^*LT CIMT* Left carotid intima-media thickness

^γ^ CIMT is the average of RT CIMT and LT CIMT

* indicates significance

There was no difference in MHR or CIMT levels between the two groups. In addition, stratified by sex, the median (IQR) of MHR values in diabetes and non-diabetic male groups were 2.42 (1.75–4.11) and 3.39 (2.36–5.09), respectively, demonstrating a difference between the two groups, with diabetic men having higher values (*p*-value = 0.035). However, there was no difference between the DM and non-DM female groups, with 2.71 (2.09–5.05) vs. 2.90 (1.96–4.76) (p-value = 0.962).

**Fig. 1:**
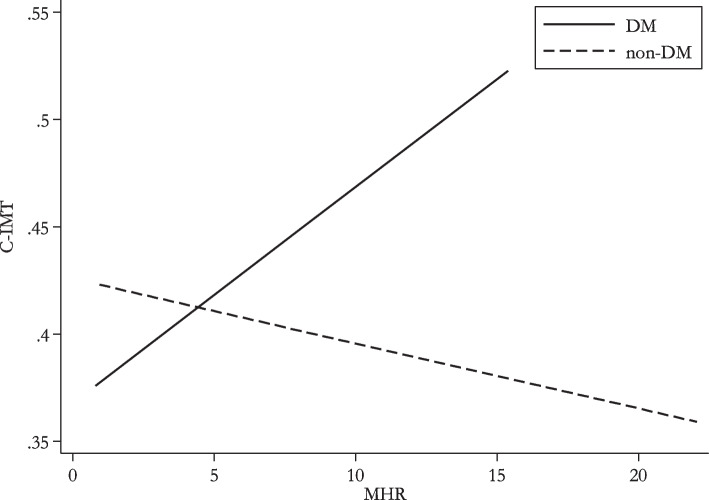
Association of carotid intima-media thickness (CIMT) and monocyte/high-density lipoprotein cholesterol ratio (MHR)

Figure 1 shows the linear relationship between MHR and CIMT in both DM and non-DM patients. Furthermore, the Spearman correlations between MHR and CIMT (*p*-values = 0.001 and 0.379, respectively) were found to be 0.32 and − 0.08 in the DM and non-DM groups, indicating that CIMT rises with higher MHR in diabetic patients compared to non-diabetics.

Finally, the regression models were built to quantify the influence of MHR on CIMT, coarsely, corrected for any of the covariates’ BMI as a clinical factor [21], age, LDL, and e-GFR (due to the group differences observed in tables 1 and 2), and completely adjusted, stratified by DM/non-DM and male/female. Table 4 summarizes the research results.

**Table 4:** Univariate and multivariate regression analysis were performed on carotid intima-media thickness

Variable	DM^†^-Female		Non-DM-Female		DM-Male		Non-DM-Male	
	Beta	*P*-value	Beta	*P*-value	Beta	*P*-value	Beta	*P*-value
Crude	0.127	0.32	−0.065	0.59	0.294	0.02*	−0.097	0.47
Age-adjusted	0.128	0.32	−0.032	0.76	0.248	0.04*	−0.093	0.46
BMI^α^ -adjusted	0.097	0.44	−0.046	0.70	0.290	0.03*	−0.082	0.54
LDL- C^β^ -adjusted	0.033	0.76	−0.081	0.49	0.282	0.03*	−0.060	0.65
e-GFR^¥^-adjusted	0.106	0.40	−0.061	0.61	0.248	0.05*	−0.081	0.54
Fully-adjusted^**^	0.028	0.80	−0.024	0.80	0.220	0.07	−0.042	0.74

*Beta* standardized Beta

^†^*DM* Diabetes mellitus

^α^*BMI* Body mass index

^β^*LDL-C* Low-density lipoprotein cholesterol

^¥^*e-GFR* Estimated glomerular filtration rate

^**^Adjusted for age, BMI, LDL-C, and e-GFR

* indicates significance

MHR was shown to be a significant predictor of CIMT in only male DM participants when crudely adjusted for confounders.

## Discussion

The most notable result of this research is that MHR and CIMT had a positive link in male T2DM patients; this correlation was not detected in non-diabetic controls.

In terms of CIMT thickening, the monocyte/HDL ratio (MHR) is preferred to the standard lipid profile alone, and it is also an independent predictor for predicting the advancement of CIMT in T2DM patients [1].

Patients with T2DM are more likely to develop micro-and macrovascular consequences of diabetes, such as atherosclerosis (AS), cardiovascular disease (CVD), and diabetic nephropathy (DN), due to a disruption in glucose and lipid metabolism (dyslipidemia), as well as a chronic inflammatory state [1–4]. Inflammation is not merely a local reaction; it may also be thought of as a systemic process that increases inflammatory mediators [3].

Monocytes have been linked to the development of diabetic micro-and macrovascular problems in people with T2DM [22, 23], and monocyte numbers have been linked to insulin resistance and coronary artery disease [24–26]. On the other hand, preliminary evidence suggests that low HDL-C contributes significantly to increased atherosclerosis in diabetes individuals [27].

As a result, the combination of monocyte count and HDL, referred to as MHR, is regarded to be a superior predictive factor concerning vascular structural change in diabetes patients than either variable alone, as indicated in the current research. The monocyte/HDL ratio is a new and simple metric that is associated with inflammatory and oxidative stress [11]. Increased MHR has been correlated with diabetic kidney disease, and so might be employed as a diabetic nephropathy biomarker [13].

Renal impairment is also associated with a higher circulating monocyte count and a lower HDL-C content in the blood, as well as more severe AS [14]. Furthermore, diabetes mellitus is the most frequent cause of CKD in the United States and across the globe, with the majority of CKD patients diagnosed at an early stage (stage 1 or 2) [15].

We showed that MHR has a greater effect in predicting subclinical and asymptomatic carotid atherosclerosis in diabetes individuals in our research. In our investigation, no differences in MHR and CIMT values were found between the two diabetic and non-diabetic groups; however, when these variables were stratified by sex, the MHR values in the DM and non-DM male groups were greater in male diabetics.

In addition, there was a direct association between MHR and CIMT in the DM group, while no such link was found in the non-DM patients. Chen et al. performed research with 494 diabetic and 1848 non-diabetic patients and found that individuals with T2DM had a stronger connection between MHR and CIMT than non-diabetic controls [1].

Karatas et al. also discovered that patients with diabetic nephropathy had greater MHR, which was more noticeable in macroalbuminuric diabetic patients, however, MHR was not observed to be higher in non-nephropathic diabetic patients as compared to healthy persons [13]. After adjusting for age and sex, we discovered that non-diabetic patients had a higher MHR than diabetic patients, but the difference was not statistically significant (*p* value = 0.13). Although the predictive significance of changes in CIMT rate in assessing cardiovascular risk remains equivocal, increased CIMT is related to future cerebrovascular and cardiovascular events [16, 28, 29].

CIMT may be utilized as a noninvasive evaluation and a substitute end goal for cardiovascular disease in individuals with T2DM, given the influence of chronic inflammation on AS, which develops most often in DM. The Spearman correlations between MHR and CIMT in the present research were 0.32 and − 0.08 in the diabetic and non-diabetic groups, respectively (*p*-values = 0.001 and 0.379), indicating that CIMT rises with increased MHR values in diabetes patients compared to non-diabetics.

The goal of this research was to show a link between MHR and CIMT in T2DM patients, even though, similar to Chen et al., the MHR may predict the CIMT by paying attention to univariate and multivariate regression analysis [1].

## Limitations

In our research, we came across several restrictions. First, because of the COVID-19 pandemic and a lower number of patients referred to the research, obtaining data and enrolling cases were very challenging. Second, due to the small sample size of our investigation, we were unable to assess the relationship between CIMT advancement and MHR in T2DM patients. Third, MHR was not examined dynamically; therefore the relationship between MHR changes and CIMT development is unknown. Therefore additional research with a larger sample size is needed to see whether lowering MHR slows down the course of atherosclerosis.

## Conclusions

In conclusion, our research demonstrates that MHR is a good and accurate predictor of the existence and development of subclinical carotid atherosclerosis in T2DM patients, particularly in men. It was also discovered that there is a direct link between MHR and CIMT in diabetes mellitus. In addition, in male diabetic subjects, MHR was demonstrated to be a predictor of CIMT.

## Data Availability

The datasets used and/or analyzed during the current study are available from the corresponding author upon reasonable request.

## Abbreviations

AS: Atherosclerosis
BMI: Body mass index
BP: Blood pressure
CBC: Complete blood count
CCA: Common carotid artery
CIMT: Carotid intima-media thickness
CKD: Chronic kidney disease
CV: Cardiovascular
CVD: Cardiovascular disease
DM: Diabetes mellitus
e-GFR: Estimated glomerular filtration rate
FBS: Fasting blood sugar
HbA1C: Hemoglobin A1C
HDL-C: High-density lipoprotein cholesterol
LDL-C: Low-density lipoprotein cholesterol
MHR: Monocyte/high-density lipoprotein cholesterol ratio
